# Ergosteryl 2-naphthoate, An Ergosterol Derivative, Exhibits Antidepressant Effects Mediated by the Modification of GABAergic and Glutamatergic Systems

**DOI:** 10.3390/molecules22040565

**Published:** 2017-03-31

**Authors:** Mingzhu Lin, Haijun Li, Yan Zhao, Enbo Cai, Hongyan Zhu, Yugang Gao, Shuangli Liu, He Yang, Lianxue Zhang, Guosheng Tang, Ruiqing Wang

**Affiliations:** 1 College of Chinese Medicinal Materials, Jilin Agriculture University, Xincheng Street 2888th, Changchun 130118, Jilin, China; 18037179167@163.com (M.L.); 18339901371@163.com (E.C.); popzhy@163.com (H.Z.); gaoyugang_2006@163.com (Y.G.); Liu-shuangli@163.com (S.L.); 15543098032@163.com (H.Y.); zlxbooksea@163.com (L.Z.); tangguosheng0113@126.com (G.T.); 2 Institute of Translational Medicine, the First Hospital of Jilin University, Xinmin Street 71th, Changchun 130021, Jilin, China; hjli2012@jlu.edu.cn; 3 Center of Ophthalmology, the Second Hospital of Jilin University, Ziqiang Street 218th, Changchun 130041, Jilin, China; m15038326367@163.com

**Keywords:** ergosterol, ergosteryl 2-naphthoate, antidepressant, forced swim test

## Abstract

Phytosterols are a kind of natural component including sitosterol, campesterol, avenasterol, ergosterol (Er) and others. Their main natural sources are vegetable oils and their processed products, followed by grains, by-products of cereals and nuts, and small amounts of fruits, vegetables and mushrooms. In this study, three new Er monoester derivatives were obtained from the reflux reaction with Er: organic acids (furoic acid, salicylic acid and 2-naphthoic acid), 1-Ethylethyl-3-(3-dimethyllaminopropyl) carbodiimide hydrochloride (EDCI) and 4-dimethylaminopyridine (DMAP) in dichloromethane. Their chemical structures were defined by IR and NMR. The present study was also undertaken to investigate the antidepressant-like effects of Er and its derivatives in male adult mice models of depression, and their probable involvement of GABAergic and glutamatergic systems by the forced swim test (FST). The results indicated that Er and its derivatives display antidepressant effects. Moreover, one derivative of Er, ergosteryl 2-naphthoate (ErN), exhibited stronger antidepressant activity in vivo compared to Er. Acute administration of ErN (5 mg/kg, i.p.) and a combination of ErN (0.5 mg/kg, i.p.), reboxetine (2.5 mg/kg, i.p.), and tianeptine (15 mg/kg, i.p.) reduced the immobility time in the FST. Pretreatment with bicuculline (a competitive γ-aminobutyric acid (GABA) antagonist, 4 mg/kg, i.p.) and *N*-methyl-d-aspartic acid (NMDA, an agonist at the glutamate site, 75 mg/kg, i.p.) effectively reversed the antidepressant-like effect of ErN (5 mg/kg, i.p.). However, prazosin (a α1-adrenoceptor antagonist, 1 mg/kg, i.p.) and haloperidol (a non-selective D2 receptor antagonist, 0.2 mg/kg, i.p.) did not eliminate the reduced immobility time. Altogether, these results indicated that ErN produced antidepressant-like activity, which might be mediated by GABAergic and glutamatergic systems.

## 1. Introduction

Depression, a prevalent psychiatric disorder with symptoms of sadness, loss of interest and appetite, fatigue, low self-esteem, inattention, insomnia and recurrent thoughts of death or suicide, is considered as one of the leading public health problem and has a lifetime prevalence of approximately 15%~17% in the world today [1,2,3]. Moreover, patients with major depression have symptoms that reflect changes in brain monoamine neurotransmitters, specifically norepinephrine (NA), GABA, serotonin (5-HT) and dopamine (DA) [4]. The exact mechanism of depression remains obscure. However, the monoaminergic hypothesis is the currently accepted mechanism of depression. Monoaminergic neurotransmitters exert major depletion in the central nervous system (CNS) [5].

Currently, the most widely used antidepressant drugs are chemical synthetic drugs, comprising monoamine oxidase inhibitors (MAOIs), selective serotonin reuptake inhibitors (SSRIs), selective noradrenaline reuptake inhibitors (SNRIs) and tricyclic antidepressants (TCAs). Most of these often generate severe side-effects and can only ameliorate some depressive symptoms [6,7]. Therefore, exploring novel, efficient potential drugs with low side-effects from natural medicine has generated considerable interest for more and more investigators.

Phytosterols are a kind of natural component including sitosterol, campesterol, avenasterol, ergosterol (Er) and others. Their main natural sources are vegetable oils and their processed products, followed by grains, by-products of cereals and nuts, and small amounts of fruits, vegetables and mushrooms. Antidepressant-like effects, their mechanism, and neurotransmitter systems involved in such antidepressant effects of phytosterols have not been investigated.

In this paper, the antidepressant-like activities of Er and its derivatives were investigated, and the mechanism and characters of the antidepressant-like actions of ergosteryl 2-naphthoate (ErN) were also explored on different CNS functions, including GABAergic and glutamatergic systems, in the forced swim test (FST).

## 2. Methods and Materials

### 2.1. Materials and Agents

Er, furoic acid, 2-naphthoic acid, salicylic acid, 4-dimethylaminopyridine (DMAP) and 1-Ethylethyl-3-(3-dimethyllaminopropyl) carbodiimide hydrochloride (EDCI) were purchased from Sigma (St. Louis, MO, USA).

Antidepressant drugs: fluoxetine (5 mg/kg, 20 mg/kg, an antidepressant drug belonging to SSRIs), tianeptine (15 mg/kg, an antidepressant drug belonging to TCAs) and reboxetine (2.5 mg/kg, an antidepressant drug belonging to NA reuptake inhibitors); and receptor antagonists and agonist: prazosin (1 mg/kg, a α1-adrenoceptor antagonist), haloperidol (0.2 mg/kg, a non-selective D2 receptor antagonist), bicuculline (4 mg/kg, a competitive GABA antagonist) and *N*-methyl-d-aspartic acid (NMDA, 75 mg/kg, an agonist at the glutamate (Glu) site) were also from Sigma. GABA and Glu standards were from Sigma, and the purity of these two standards was over 98% as indicated by the manufacturer. All other reagents used in the study were of analytical grade.

SPF (specific pathogen free) grade, male Institute of Cancer Research (ICR) mice (18–22 g) were obtained from Changchun Yisi experimental animal technology Co., Ltd. (Changchun, China). The mice were kept in the laboratory under the following conditions: 23 ± 2 °C, 55% ± 5% humidity on a 12-h light/dark cycle. The mice were provided with a standard pellet diet and water *ad libitum* during the experimental period. After one week of acclimatization, all mice were randomly divided into different groups (*n* = 10).

### 2.2. Synthesis of Compounds

To a solution of 2-naphthoic acid (0.3 mmol) and EDCI (0.4 mmol) in 5 mL dichloromethane stired for 10 minutes, we added a solution of 0.2 mmol of ER and DMAP (0.2 mmol) in 5 mL dichloromethane. After the solution was heated to reflux for 8 h, the precipitate was removed by filtration, and the solvent was removed under reduced pressure. The residue was purified by silica gel chromatography and eluted with petroleum ether/ethyl acetate (5:1, *v*/*v*) to yield the product –ErN as a light white solid (89.76 mg). The purity of ErN was determined as 97.3% by HPLC, and its structure was established by IR and NMR analysis. Other Er derivatives (ergosteryl furan-2-carboxylate (ErF) and ergosteryl 2-hydroxybenzoate (ErH)) were prepared by the same methods.

### 2.3. Structural Determination

The molecular structures of Er and its derivatives were identified by IR and NMR. NMR spectra were recorded on a Varian Mercury 300 MHz NMR spectrometer equipped with an Oxford Instruments Ltd. superconducting magnet (Palo Alto, CA, USA). CDCl_3_ was used as a solvent to dissolve samples and tetramethylsilane was used as the internal standard for NMR analysis. FTIR analysis was performed in a WGH-30A double-beam infrared spectrophotometer (Gangdong Sci. & Tech. development Co., Ltd. Tianjin, China).

### 2.4. Spontaneous Locomotor Activity Test (SLT)

In order to exclude the possibility that the alteration in the immobility time in the FST was due to interference of the locomotor activity, spontaneous locomotor activity of each mouse was observed in a ZZ-6 mouse autonomic activity test instrument (Shanghai benefits of the medical equipment Development Co., Ltd., Shanghai, China). The apparatus was placed in a sound attenuated testing room. The times of autonomous activities were evaluated over a 5-min period [8,9]. Each animal was used only once.

### 2.5. FST

Mice were forced to swim individually in a glass jar (height 25 cm, 14 × 30 cm^2^), containing 15 cm of fresh water at 23–25 °C. After an initial 2-min period of vigorous activity, each animal assumed a typical immobile posture. A mouse was considered to be immobile when it remained floating in the water without struggling, making only minimum movements of its limbs necessary to keep its head above water. The total duration of immobility was recorded during the next 4 min of a total 6-min test [10]. The changes in immobility duration were studied after administering drugs in separate groups of animals. Each animal was used only once [11,12,13].

### 2.6. Drug Treatment

The tested materials, that is, Er and its derivatives, were dissolved in 40% propylene glycol, whereas all the other drugs were dissolved in isotonic saline solution (0.9% NaCl aq) immediately before use. The mice of the negative control group were treated for the same volume of 40% propylene glycol. All the tested mice were administered by i.p. route in the SLT and FST. The administration operators were blind to the drugs including the vehicles, the tested materials, antidepressant drugs and receptor antagonists and agonists. The observers were also blind to the drug treatment. Specific tests were arranged as follows.

#### 2.6.1. The Antidepressant Effect of Er and Its Derivatives in the FST

Propylene glycol and the tested drugs including Er, ErF, ErS and ErN (5 mg/kg and 20 mg/kg, respectively) were injected and the immobility period was recorded after 60 min of administration.

#### 2.6.2. The Effective and Sub-Effective Doses of ErN in the FST

Positive drug groups (sub-effective doses): fluoxetine (5 mg/kg), tianeptine (15 mg/kg) and reboxetine (2.5 mg/kg) were administered 60 min prior to the FST. ErN groups: ErN (0.1 mg/kg, 0.5 mg/kg, 1 mg/kg, and 5 mg/kg, respectively) was administered 60 min before the immobility period was recorded. Positive drug + ErN groups: fluoxetine (5 mg/kg), tianeptine (15 mg/kg) and reboxetine (2.5 mg/kg) were administered immediately after administration of ErN (0.5 mg/kg) 60 min prior to the experiment.

#### 2.6.3. The Effect of ErN on Locomotor Activity

Propylene glycol and ErN (0.5 mg/kg and 5 mg/kg) were injected and the autonomous activity in the SLT was recorded after 60 min of administration, respectively.

#### 2.6.4. The Roles of Different CNS Functions Including Noradrenergic, Dopaminergic, GABAergic and Glutamatergic Systems in the Antidepressant-Like Effect of ErN in the FST

Prazosin group: administered prazosin (1 mg/kg) 60 min prior to the experiment. ErN (5 mg/kg) + prazosin (1 mg/kg) group: administered prazosin 30 min prior to ErN, and performed behavioral experiment after 60 min.

Bicuculline group: administered bicuculline (4 mg/kg) 60 min prior to the experiment. ErN (5 mg/kg) + bicuculline (4 mg/kg) group: administered bicuculline 30 min prior to ErN, and performed behavioral experiment after 60 min.

Haloperidol group: administered haloperidol (0.2 mg/kg) 60 min prior to the experiment. ErN (5 mg/kg) + haloperidol (0.2 mg/kg) group: administered haloperidol 30 min prior to ErN, and performed behavioral experiment after 60 min.

NMDA group: administered NMDA (75 mg/kg) 60 min prior to the experiment. ErN (5 mg/kg) + NMDA (75 mg/kg) group: administered NMDA 30 min prior to ErN, and performed behavioral experiment after 60 min.

### 2.7. Biochemical Measurements

The mice brain was washed with ice-cold physiological saline and homogenized, shaken for 10 s and centrifuged at 12,000× *g* for 5 min at 4 °C. The supernatants were collected for the detection of GABA and Glu by RP-HPLC method [14]. The samples were pre-column derivatization with 2,4-dinitrofluorobenzene (DNFB). The content was calculated by external standard method. HPLC analysis was carried out on a Shimadzu LC2010A series HPLC system (Shimadzu, Kyoto, Japan). An Agilent C_18_ column (200 mm × 4.6 mm, i.d., 5 μm) was used for all separations at a column temperature of 35 °C. The binary gradient elution system consisted of 0.05 mol/L sodium acetate buffer (pH 6.0, A) and Acetonitrile/water (1:1, *v*/*v*, B). The separation was achieved using the following gradient program: 0–10 min (16%–45% B), 10–18 min (85% B). The flow-rate was set at 1.0 mL/min and the sample injection volume was 20 μL. The signals were detected by UV detector at 350 nm.

### 2.8. Statistical Analysis

All values were expressed as mean ± S.D. The data were statistically analyzed using the *t*-test and one-way analyses of variance (ANOVAs), followed by Tukey’s *post-hoc* multiple comparison test. A value of *p* < 0.05 was considered statistically significant.

## 3. Results

### 3.1. Synthesis and Structural Identification

**Figure 1:**
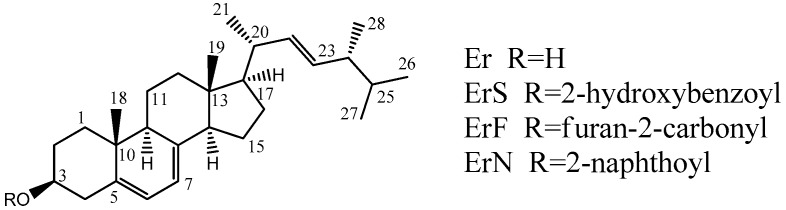
Structures of compounds.

The Er esterification derivatives (shown in Figure 1) were obtained via the reaction of Er with organic acids in dichloromethane at a temperature of 70 °C by the way of reflux. Because water produced during the reaction slows down or even stops the reaction, we carried out the reversible process with two molar ratios of EDCI to avoid side-effects. DMAP was used as a catalyst. The products were isolated using a silica gel column. Two methods (FTIR and NMR) were used to identify the molecular structures of the synthesized Er esters. The IR absorption spectrum of Er shows an absorption at 1710.7 cm^−1^, which is from C=O stretch, and the absorption bands at 3342.0–3401.7 cm^−1^ indicate the existence of -O-H (-O-H stretch). For all Er esters, absorption was observed at 1674–1730 cm^−1^ (C=O stretch in moiety of acyl group), indicating the introduction of an acyl group. The molecular structures of synthesized products were identified by individual NMR analysis, and the characteristic chemical shifts were detailed as follows. In ^1^H-NMR spectrum of all Er esters, the signal at 3.0–6.0 (*s*, 1H) disappears, indicating -OH was esterified. The molecular structures of synthesized products were also identified by individual ^13^C-NMR analysis, and the characteristic chemical shifts were detailed as follows. In ^13^C-NMR spectrum of all Er esters, the signal at 71.6 ppm disappears and reappears at about 72.00–74.40 ppm, indicating -OH was esterified, while the signals at 157.18–168.62 ppm indicate the introduction of C=O. Compared with ^1^H-NMR, ^13^C-NMR shows that the esterification reaction was successful.

#### 3.1.1. ErN

Yield 79.01%, white powder, C_39_H_50_O_2_. IR: 3083, 2985–2875, 1725, 1603, 1582, 1505, 1383, 1371 cm^−1^; ^1^H-NMR (300 MHz, CDCl_3_) *δ*ppm: 8.618 (*s*, 1H, 1″-H), 8.102 (*dd*, 1H, *J* = 1.5, 8.7 Hz, 8″-H), 7.973 (*dd*, 1H, *J* = 1.2, 8.7 Hz, 5″-H), 7.890 (*d*, 2H, *J* = 8.7 Hz, 3″,4″-H), 7.611 (*ddd*, 1H, *J* = 1.2, 8.7, 14.4 Hz, 6″-H), 7.588 (*ddd*, 1H, *J* = 1.5, 8.7, 14.4 Hz, 7″-H), 5.649 (*m*, 1H, 6-H), 5.427 (*m*, 1H, 7-H), 5.240 (*dd*, 1H, *J* = 4.2, 7.2 Hz, 22-H), 5.221 (*dd*, 1H, *J* = 4.2, 7.2 Hz, 23-H), 5.043 (*m*, 1H, 3-H), 1.075 (*d*, 3H, *J* = 6.6 Hz, 21-H), 1.031 (s, 3H, 18-H), 0.956 (*d*, 3H, *J* = 6.6 Hz, 28-H), 0.877 (*d*, 3H, *J* = 6.9 Hz, 26-H), 0.862 (*d*, 3H, *J* = 6.6 Hz, 27-H), 0.657 (*s*, 3H, 19-H), 2.754~0.573 (others); ^13^C-NMR (75 MHz, CDCl_3_) *δ*ppm: 165.05 (C1′), 140.47 (C8), 137.45 (C5), 134.52 (C22), 134.41 (C4a″), 131.44 (C8a″), 130.91 (C23), 129.86 (C1″), 128.26 (C8″), 127.06 (C6″), 126.98 (C4″), 126.93 (C5″), 126.68 (C2″), 125.50 (C7″), 124.24 (C3″), 119.30 (C6), 115.31 (C7), 72.50 (C3), 54.64 (C17), 53.47 (C14), 45.00 (C9), 41.78 (C13,C24), 39.42 (C20), 37.98 (C12), 36.94 (C1), 36.11 (C10), 35.78 (C4), 32.04 (C26), 27.26 (C2), 27.21 (C16), 21.96 (C15), 20.09 (C11), 19.99 (C26), 18.94 (C27), 18.63 (C21), 16.59 (C28), 15.19 (C18), 11.02 (C19).

#### 3.1.2. ErF

Yield 85.23%, white powder, C_33_H_46_O_3_. IR: 3052, 2972-2884, 1730, 1652, 1384, 1370 cm^−1^; ^1^H-NMR (300 MHz, CDCl_3_) *δ*ppm: 7.504 (*dd*, 1H, *J* = 0.9, 1.8 Hz, 5″-H), 7.111 (*dd*, 1H, *J* = 0.9, 3.6 Hz, 3″-H), 6.441 (*dd*, 1H, *J* = 1.8, 3.6 Hz, 4″-H), 5.542 (*m*, 1H, 6-H), 5.334 (*m*, 1H, 7-H), 5.151 (*dd*, 1H, *J* = 4.2, 7.2 Hz, 22-H), 5.137 (*dd*, 1H, *J* = 4.2, 7.2 Hz, 23-H), 4.883 (*m*, 1H, 3-H), 0.982 (*d*, 3H, *J* = 6.6 Hz, 21-CH_3_), 0.916 (*s*, 3H, 18-H), 0.860 (*d*, 3H, *J* = 6.9 Hz, 28-H), 0.781 (*d*, 3H, *J* = 6.9 Hz, 26-H), 0.766 (*d*, 3H, *J* = 6.9 Hz, 27-H), 0.565 (*s*, 3H, 19-H), 2.588~0.479; ^13^C-NMR (75 MHz, CDCl_3_) *δ*ppm: 157.18 (C1′), 145.07 (C5″), 144.06 (C2″), 140. 60 (C8), 137.28 (C5), 134.54 (C22), 130.96 (C23), 119.38 (C6), 116.68 (C3″), 115.28 (C7), 110.74 (C4″), 72.53 (C3), 54.69 (C17), 53.50 (C14), 45.02 (C9), 41.80 (C13,24), 39.42 (C20), 38.00 (C12), 36.90 (C1), 36.10 (C4), 35.67 (C10), 32.07 (C25), 27.26 (C2), 27.15 (C16), 21.97 (C15), 20.09 (C11), 20.01 (C26), 18.93 (C27), 18.63 (C21), 16.59 (C28), 15.16 (C18), 11.04 (C19).

#### 3.1.3. ErS

Yield 87.85%, light yellow powder, C_35_H_48_O_3_. IR: 3440, 3080, 2974-2875, 1674, 1604, 1582, 1382, 1370 cm^−1^; ^1^H-NMR (300 MHz, CDCl_3_) *δ*ppm: 10.904 (*s*, 1H, 2″-OH), 7.871 (*dd*, 1H, *J* = 1.5, 7.8 Hz, 6″-H), 7.477 (*dt*, 1H, *J* = 1.8, 7.8 Hz, 4″-H), 6.990 (*d*, 1H, *J* = 7.8 Hz, 3″-H), 6.904 (*dt*, 1H, *J* = 0.9, 7.8 Hz, 5″-H), 5.637 (*m*, 1H, 6-H), 5.421 (*m*, 1H, 7-H), 5.281 (*dd*, 1H, *J* = 4.2, 7.2 Hz, 22-H), 5.140 (*dd*, 1H, *J* = 4.2, 7.2 Hz, 23-H), 5.029 (*m*, 1H, 3-H), 1.062 (*d*, 3H, *J* = 6.6 Hz, 21-CH_3_), 1.009 (*s*, 3H, 18-H), 0.940 (*d*, 3H, *J* = 6.9Hz, 28-H), 0.860 (*d*, 3H, *J* = 6.6 Hz, 26-H), 0.845 (*d*, 3H, *J* = 6.6 Hz, 27-H), 0.649 (*s*, 3H, 19-H), 2.672~0.562. ^13^C-NMR (75 MHz, CDCl_3_) *δ*ppm: 168.62 (C1′), 160.70 (C2″), 140.71 (C8), 136.99 (C5), 134.51 (C22, C4″), 130.98 (C23), 128.86 (C6″), 119.56 (C5″), 117.98 (C6), 116.51 (C3″), 115.27 (C7), 111.80 (C1″), 73.08 (C3), 54.69 (C17), 53. 52 (C14), 45.02 (C9), 41.80 (C13,24), 39.40 (C20), 37.99 (C12), 36.85 (C1), 36.11 (C10), 35.60 (C4), 32.07 (C25), 27.27 (C2), 27.11(C16), 21.98 (C15), 20.10 (C11), 20.03 (C26), 18.94 (C27), 18.64 (C21), 16.60 (C28), 15.19 (C18), 11.06 (C19).

### 3.2. The Antidepressant Effect of Er and Its Derivatives in the FST

**Figure 2:**
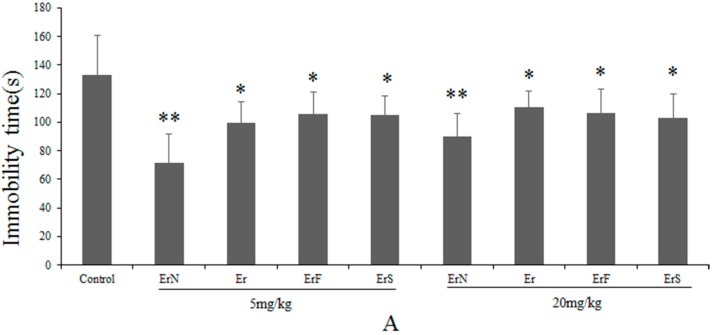

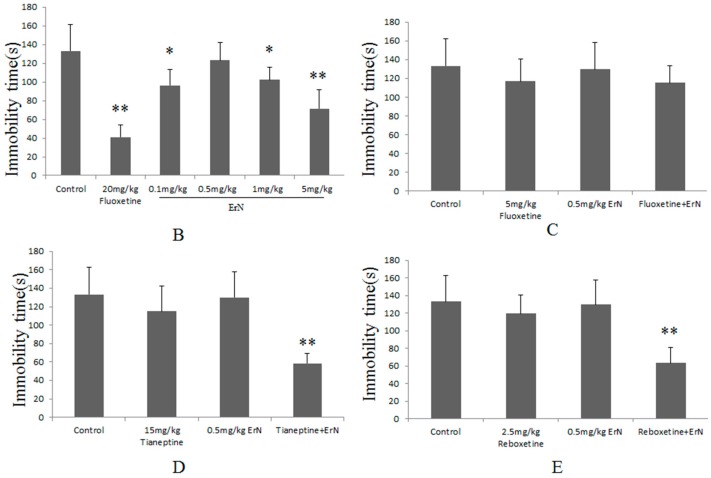
(**A**) shows the antidepressant-like effect of different doses of Er and its derivatives in the FST; (**B**) shows the antidepressant-like effect of different doses of ErN in the FST; (**C**) shows the antidepressant-like effect of the co-administration of sub-effective doses of ErN (0.5 mg/kg, i.p.) with fluoxetine (5 mg/kg, i.p.) in the FST; (**D**) shows the antidepressant-like effect of the co-administration of sub-effective doses of ErN (0.5 mg/kg, i.p.) with tianeptine (15 mg/kg, i.p.) in the FST; (**E**) shows the antidepressant-like effect of the co-administration of sub-effective doses of ErN (0.5 mg/kg, i.p.) with reboxetine (2.5 mg/kg, i.p.) in the FST. The values represent the mean ± SD (*n* = 10 in each group), compared with the control group, * *p* < 0.05, ** *p* < 0.01. One way ANOVA, *post-hoc* Tukey test.

In the process of antidepressant screening, we found that Er and its derivatives exhibit a certain antidepressant activity in the mouse model of depression. Furthermore, the optimum dose of Er and its derivatives was screened over a wide range (0.1 mg/kg–100 mg/kg), and the results showed that Er and its derivatives in a dosage of 1 mg/kg–20 mg/kg could significantly reduce the immobility time in the FST compared with the control group. The antidepressant-like activities of Er and its derivatives in vivo were shown in Figure 2A. The results indicated that the immobility time was decreased in mice treated with Er and its derivatives; furthermore, ErN showed the lowest immobility time. Following, ErN continued to be subjected to subsequent experiments.

### 3.3. The Effective and Sub-Effective Doses of ErN in the FST

Figure 2B shows that ErN (5 mg/kg, i.p.) (F(1,18) = 22.57, *p* < 0.01) significantly reduced the immobility time in the FST compared with other dose groups, and the dosage of 0.5 mg/kg could not reduce the immobility time compared with that of the control group. Therefore, 5 mg/kg and 0.5 mg/kg were chosen as the effective dose and sub-effective dose, respectively.

### 3.4. The Antidepressant Effect of Co-Administration of the Sub-Effective Doses of ErN (0.5 mg/kg) and Antidepressant Drugs in the FST

The results of co-administration in the FST were shown in Figure 2C–E. Figure 2C presented that the co-administration of sub-effective fluoxetine (5 mg/kg, i.p.) and ErN (0.5 mg/kg, i.p.) could not reduce the immobility time compared with that of the control group in the FST. The *t*-test showed there was no difference for ErN treatment, fluoxetine treatment and ErN combined with fluoxetine.

The results presented in Figure 2D showed that the concurrent administration of the sub-effective dose of tianeptine (15 mg/kg, i.p.) and ErN (0.5 mg/kg, i.p.) (F(1,18) = 56.80, *p* < 0.01) led to a significantly shorter immobility time compared with either of those substances treated alone as well as the control group. The *t*-test indicated significant effects of drug treatment on the immobility time in the FST for animals treated with the sub-effective doses of tianeptine and ErN interaction; it also revealed that neither tianeptine nor ErN alone reduced the immobility time in the FST compared to that of the control group.

The effect of a co-administration of the sub-effective doses of reboxetine (2.5 mg/kg, i.p.) and ErN (0.5 mg/kg, i.p.) (F(1,18) = 41.81, *p* < 0.01) on the immobility time in the FST was shown as in Figure 2E. The sub-effective dose of ErN and reboxetine interaction considerably reduced the immobility time in the FST compared to either of those treated alone as well as the control group.

### 3.5. The Roles of Different the CNS Functions Including Noradrenergic, Dopaminergic, GABAergic and Glutamatergic Systems in the Antidepressant-Like Effect of ErN (5 mg/kg) in the FST

**Figure 3:**
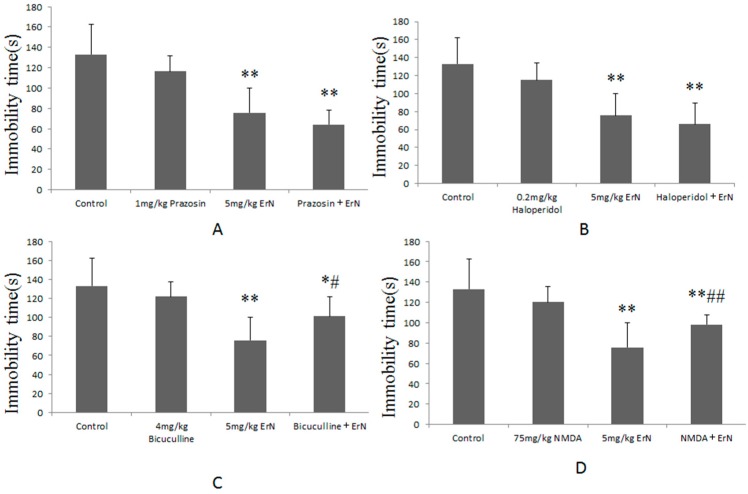
(**A**) shows effects of pre-treatment with prazosin (1mg/kg, i.p.) on the antidepressant-like effect induced by ErN (5 mg/kg, i.p.) in the FST; (**B**) shows effects of pre-treatment with haloperidol (0.2 mg/kg, i.p.) on the antidepressant-like effect induced by ErN (5 mg/kg, i.p.) in the FST; (**C**) shows effects of pre-treatment with bicuculline (4 mg/kg, i.p.) on the antidepressant-like effect induced by ErN (5 mg/kg, i.p.) in the FST; (**D**) shows effects of pre-treatment with NMDA (75 mg/kg, i.p.) on the antidepressant-like effect induced by ErN (5 mg/kg, i.p.) in the FST. The values represent the mean ± SD (*n* = 10 in each group), compared with control group, * *p* < 0.05, ** *p* < 0.01; compared with ErN group, ^#^ *p* < 0.05, ^##^ *p* < 0.01. One way ANOVA, *post-hoc* Tukey test.

The results of the involvement of the noradrenergic system in the antidepressant-like effect of ErN in the FST were shown in Figure 3A. It showed that the prazosin + ErN group (F(1,18) = 45.37, *p* < 0.01) was significantly different compared with the control group, but there was no significant difference compared with the ErN group. The results revealed that the pre-treatment with prazosin (1 mg/kg, i.p., α1-adrenoceptor antagonist) did not reverse the antidepressant-like effect of ErN (5 mg/kg, i.p.) in the FST.

Figure 3B showed the results of the role of the dopaminergic system in the antidepressant-like effect of ErN in the FST. The results showed that the pre-treatment with haloperidol (0.2 mg/kg, i.p., a non-selective D2 receptor antagonist) did not eliminate the antidepressant-like effect of ErN (5 mg/kg, i.p.) in the FST.

As shown in Figure 3C, the results of the involvement of the GABAergic system in the antidepressant-like effect of ErN in the FST displayed that bicuculline (4 mg/kg, i.p., a competitive GABA antagonist) significantly increased the immobility time reduced by ErN (5 mg/kg, i.p.) in the FST.

As shown in Figure 3D, the results of the involvement of the glutamatergic system in the antidepressant-like effect of ErN in the FST revealed that NMDA (75 mg/kg, i.p. an agonist at the glutamate site) considerably eliminated the antidepressant-like effect elicited by ErN (5 mg/kg, i.p.).

### 3.6. Effects of ErN on the Levels of GABA and Glu in Mice Exposed to the FST

**Figure 4:**
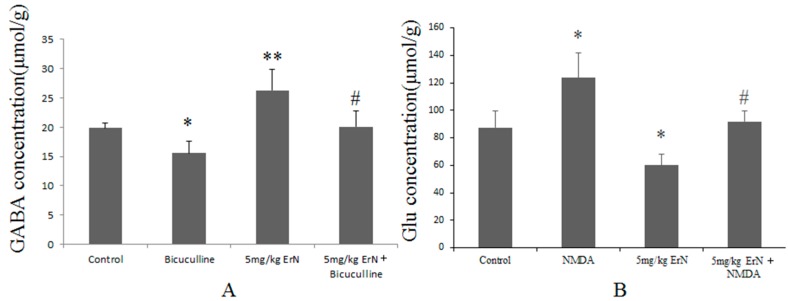
Effects of ErN, bicuculline, NMDA and co-treatment on the GABA (**A**) and Glu (**B**) levels in the brains of mice exposed to the FST. The values represent the mean ± SD (*n* = 10 in each group), compared with control group, * *p* < 0.05, ** *p* < 0.01; compared with ErN (5 mg/kg) group, ^#^ *p* < 0.05. One way ANOVA, *post-hoc* Tukey test.

The effects of ErN, bicuculline, NMDA and co-treatment on the GABA and Glu levels in the brains of mice exposed to the FST were shown in Figure 4. The GABA levels showed significant differences between the ErN group (F(1,18) = 7.61, *p* < 0.05) or bicuculline group (F(1,18) = 25.59, *p* < 0.01) and the control group; moreover, the co-treatment group showed little difference compared to the ErN group (F(1,18) = 23.97, *p* < 0.01) (Figure 4A). In Figure 4B, the ErN (F(1,18) = 7.67, *p* < 0.05) significantly increased the Glu levels compared to that of the control group; conversely, the Glu levels in the mice treated with NMDA (F(1,18) = 7.09, *p* < 0.05) was significantly decreased. Meanwhile, the co-treatment group (F(1,18) = 15.71, *p* < 0.05) exhibited a significant difference compared to the ErN group.

### 3.7. Effect on Locomotor Activity

To exclude the false positive effect in behavioral despair tests, the effect of ErN on autonomous activity in mice were tested. The results showed that there was no significant effect on the locomotor activity of mice (97 ± 10, 99 ± 11 times) when treated with ErN (5 mg/kg, 0.5mg/kg, i.p.) as compared to the control (109 ± 12 times). The results indicated that ErN did not affect the spontaneous activity of mice.

## 4. Discussion

In the present study, three new Er monoester derivatives were obtained from the reflux reaction with Er; organic acids (furoic acid, salicylic acid and 2-naphthoic acid), EDCI and DMAP in dichloromethane. Their chemical structures were defined by IR and NMR.

The FST is the most widely used animal model for evaluating pharmacological antidepressant activity [6,7] and screening new antidepressant drugs [15,16], in which mice are placed under a stressful environment and become immobile after an initial period of struggling, which is similar to human depression and is feasibly reversed by antidepressant drugs. In the present study, the results revealed that Er and its derivatives exhibited antidepressant-like effects, and that ErN was the most effective one of these derivatives. However, drugs enhancing motor activity may give a ‘false’ positive effect in the FST model [17]. Therefore, in order to exclude the possibility that the decrease of immobility time elicited by a drug in the FST was due to an enhancement by the locomotor activity, the mice were subjected to the locomotor activity test [9]. The results of this study showed that acute treatment with ErN (0.5 mg/kg and 5 mg/kg, i.p.) did not change the locomotor activity of mice.

Many studies demonstrate that the concurrent treatment with commonly used antidepressant drugs might produce a better treatment effect or faster onset speed than monotheraphy [18,19,20]. In this study, we found that ErN and tianeptine, as well as ErN and reboxetine showed synergistic effects in combination, while the combination of ErN and fluoxetine did not exhibit this performance.

Previous studies indicated that the biogenic amines (NA and DA), GABA and glutamate play a pivotal role in the pathogenesis of depression and on the effects of different antidepressant drugs [21,22].

GABA is the most important inhibitory neurotransmitter in the CNS of mammals, causing the inhibition of almost all the key neurons [23]. In recent years, many studies have reported that there is a relationship between GABA dysfunction and depression, anxiety, bipolar disorder, etc.; and GABA plays a very important role in the occurrence of affective disorder diseases [24,25,26,27]. In clinical application, some drugs and mood stabilizers such as lithium salts, divalproex, and carbamazepine, used for the treatment of severe depression, can reduce the metabolism of GABA and improve the content of GABA in brain [28]. In addition, studies of depression in animal models have shown that GABA is significantly reduced in experimental animals in the chronic stress depression model, forced swimming, and other animal models; therefore, supplementation of GABA can alleviate depression-like behavior [29,30,31,32]. In this study, bicuculline (4 mg/kg, i.p.), a competitive GABA antagonist, effectively blocked the antidepressant-like effect of the ErN (5 mg/kg, i.p.).

The glutamatergic system is reported to be involved in the pathophysiology of depression; Glu is present in more than 60% of the synaptic structure in the CNS, and has an excitatory effect on almost all the neurons in the CNS [33]. The content of the extracellular Glu is essential to maintain the normal physiological process; although the content of Glu is very high in the whole CNS, a small part of it exists the outside of the cell, and once its content is too high, Glu will produce an excitatory neurotoxic effect on cells and these cells will be damaged [34]. A number of postmortem studies indicated that there were abnormalities in glutamatergic transmission in depression and a decreased level of NMDA receptors in the hippocampus [35,36]. Moreover, NMDA receptor antagonists exhibited antidepressant-like effects [37], such as ketamine, CP-101, MK-0657, Ly235959 and GLYX-13. In the present study, we used NMDA (75mg/kg, i.p.) as an excitatory amino acid agonist, and the results indicated that it was able to prevent the reduction of the immobility time induced by the ErN (5 mg/kg, i.p.) treatment in the FST, revealing the involvement of the glutamatergic system in the antidepressant-like effect of ErN.

For biogenic amines (NA and DA), they also play a pivotal role in the pathogenesis of depression and on the effects of different antidepressant drugs. But our study showed that ErN (5 mg/kg, i.p.) did not take part in the α1 system and the D2 system in FST.

In the existing literature [9,12,13,17,33], almost all of the animals in the experimental model of depression were male. Therefore, we selected male mice as subjects in the present study. But, studies also showed that the prevalence of some mental illnesses, including major depression, anxiety-, trauma-, and stress-related disorders, is higher in women than men. This could not simply be explained by socioeconomic determinants, such as income, social status, or cultural background, and more importance should be placed on sex differences in biological, pharmacokinetic, and pharmacological factors that contribute to females’ vulnerability to these mental illnesses. Particularly, a possible role of mitochondrial function, including biosynthesis, bioenergetics, and signaling, should be considered in mediating the sex differences in psychiatric disorders [38]. Thus, in our follow-up studies, including research on the antidepressant mechanisms of sterols and their derivatives, gender factors will be introduced. The preliminary experimental results also showed that ErN is distributed in the brain tissue of mice at 1 h after administration. The overall study on the pharmacokinetic and tissue distribution of ErN will be carried out in our later experiments.

In summary, three new Er monoester derivatives were obtained from the reflux reaction with Er; organic acids (furoic acid, salicylic acid and 2-naphthoic acid), EDCI and DMAP in dichloromethane, and their chemical structures were defined by IR and NMR. What’s more, the antidepressant-like effects of Er and its derivatives were described for the first time; the results of the present study showed that acute ErN treatment and sub-effective ErN co-administrated with tianeptine or reboxetine both could reduce the immobility time, demonstrating the antidepressant-like effects of ErN in the FST. The results of our current study also indicated that this antidepressant effect might be partially mediated by GABAergic and glutamatergic systems. Based on our studies, we believe that Er and its derivatives might be potential materials for drug and food development against depression. To elucidate the detailed mechanisms of different receptors in the above systems, further investigations using other behavioral paradigms such as learned helplessness, chronic unpredictable stress (CUS) and social defeat stress models will be explored in our future scientific studies.

## Abbreviations

Er	ergosterol
EDCI	1-ethyl-3-(3-dimethyllaminopropyl) carbodiimide hydrochloride
DMAP	4-dimethylaminopyridine
ErN	ergosteryl 2-naphthoate
ErF	ergosteryl furan-2-carboxylate
ErH	ergosteryl 2-hydroxybenzoate
FST	forced swim test
CNS	central nervous system
SLT	spontaneous locomotor activity test
GABA	γ-aminobutyric acid
NA	noradrenaline
DA	dopamine
Glu	glutamate
5-HT	serotonin
MAOIs	monoamine oxidase inhibitors
SSRIs	selective serotonin reuptake inhibitors
SNRIs	selective noradrenaline reuptake inhibitors
TCAs	tricyclic antidepressants
